# A novel neo‐sex chromosome in *Sylvietta brachyura* (Macrosphenidae) adds to the extraordinary avian sex chromosome diversity among Sylvioidea songbirds

**DOI:** 10.1111/jeb.14096

**Published:** 2022-09-26

**Authors:** Hanna Sigeman, Hongkai Zhang, Salwan Ali Abed, Bengt Hansson

**Affiliations:** ^1^ Department of Biology Lund University Lund Sweden; ^2^ Ecology and Genetics Research Unit University of Oulu Oulu Finland; ^3^ College of Science, University of Al‐Qadisiyah Iraq

**Keywords:** birds, genomics, Northern crombec, sex chromosomes, *Sylvietta brachyura*, Sylvioidea

## Abstract

We report the discovery of a novel neo‐sex chromosome in an African warbler, *Sylvietta brachyura* (northern crombec; Macrosphenidae). This species is part of the Sylvioidea superfamily, where four separate autosome–sex chromosome translocation events have previously been discovered via comparative genomics of 11 of the 22 families in this clade. Our discovery here resulted from analyses of genomic data of single species‐representatives from three additional Sylvioidea families (Macrosphenidae, Pycnonotidae and Leiothrichidae). In all three species, we confirmed the translocation of a part of chromosome 4A to the sex chromosomes, which originated basally in Sylvioidea. In *S. brachyura*, we found that a part of chromosome 8 has been translocated to the sex chromosomes, forming a unique neo‐sex chromosome in this lineage. Furthermore, the non‐recombining part of 4A in *S. brachyura* is smaller than in other Sylvioidea species, which suggests that recombination continued along this region after the fusion event in the Sylvioidea ancestor. These findings reveal additional sex chromosome diversity among the Sylvioidea, where five separate translocation events are now confirmed.

## Graphical abstract

We report a novel neo‐sex chromosome in northern crombec (*Sylvietta brachyura*). This species belongs to the Sylvioidea superfamily, in which a fusion between chromosome Z and 4A occurred in a common ancestor. In northern crombec, chromosome 8 have also become fused to this neo‐sex chromosome. This is the fifth autosome–sex chromosome fusion found in the Sylvioidea superfamily.

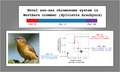

## 1 INTRODUCTION

Interchromosomal rearrangements, formed by fusions and translocations, play an important role in evolutionary processes such as reproductive isolation and speciation, by interfering with recombination and promoting genetic differentiation (Giménez et al., 2017; Kirkpatrick, 2017; Luo et al., 2018). When such fusions occur between autosomes and sex chromosomes, forming ‘neo‐sex chromosomes’, novel genes are brought into linkage with the sex determining factor. This results in contrasting evolutionary trajectories compared to their autosomal counterparts in related species and can facilitate sex‐specific selection (Bachtrog, 2020; Charlesworth, 1996; Steinemann & Steinemann, 1998; Zhou & Bachtrog, 2012). Growing evidence supports a role of neo‐sex chromosomes in sex‐specific evolution (e.g. colour patterning; Smith et al., 2016; Martin et al., 2020) and population divergence and speciation (Bracewell et al., 2017; Kitano et al., 2009).

Autosome–sex chromosome fusions were discovered through karyotype work in *Drosophila* already in the middle of the 20th century (Patterson & Stone, 1952) and have since been discovered and studied in both male (XY) and female heterogametic (ZW) systems (e.g. Nguyen et al., 2013; Rovatsos et al., 2019; Smith et al., 2016). There is a bias towards studies on XY systems, which may partly be because fusions involving the Y chromosome are more biologically widespread than fusions to X, Z and W (Pennell et al., 2015), partly because XY systems have generally been more well explored than ZW systems. The recent revolution in genome sequencing technology has resulted in a dramatic increase of detailed studies of novel sex chromosomes, including neo‐ZW systems. This is a promising development of the research field as continued characterization of autosome–sex chromosome fusions broadly among XY and ZW systems is needed to promote understanding of the causes of sex chromosome rearrangements, responses to sex‐specific selection and the evolution of reproductive isolation.

**FIGURE 1:**
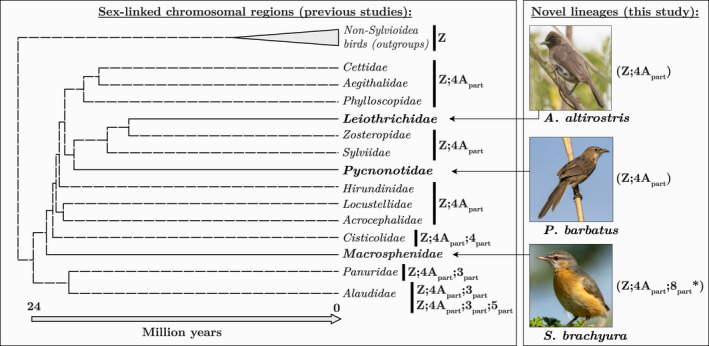
Phylogenetic relationship and chromosomal origin of sex‐linked regions among Sylvioidea families studied to date (those included in this study are in bold). Topology and branch lengths are from Oliveros et al. (2019). Information on sex‐linked chromosomal regions comes from Dierickx et al. (2020); Leroy et al. (2021); Pala, Hasselquist, et al. (2012); Pala, Naurin, et al. (2012); Sigeman et al. (2019; 2021) and this study. The translocation involving chromosome 8 (indicated with *) is a novel finding for this study.

The avian ZW sex chromosome system was long viewed as extremely stable (Nanda et al., 2008). However, recent discoveries of neo‐sex chromosomes have revealed that avian sex chromosomes are more variable than was previously believed (Burley et al., 2022; Dierickx et al., 2020; Gan et al., 2019; Huang et al., 2021; Kretschmer et al., 2020; Leroy et al., 2021; Pala, Hasselquist, et al., 2012; Pala, Naurin, et al., 2012; Sardell, 2016; Sigeman et al., 2019, 2020). The most diverse avian sex chromosomes currently known are found among members of the Sylvioidea (Dierickx et al., 2020; Leroy et al., 2021; Pala, Hasselquist, et al., 2012; Sigeman et al., 2019, 2020). This superfamily of songbirds consists of more than 1200 species that diverged from other songbirds approximately 24 million years ago (Fregin et al., 2012; Oliveros et al., 2019). Previous comparative genomic analyses covering 11 of the 22 families within Sylvioidea detected enlarged sex chromosomes formed by four independent translocation events (Figure 1). In all Sylvioidea species studied so far, a part of chromosome 4A (based on chromosome naming in *Taeniopygia guttata*, zebra finch) has been translocated to the ancestral sex chromosomes (Pala, Hasselquist, et al., 2012; Pala, Naurin, et al., 2012; Figure 1). The translocated region of chromosome 4A (0–9.6 Mbp) has fused to both chromosome Z and W, whereas the rest of the chromosome (9.6–20.7 Mbp) has remained autosomal (Ponnikas et al., 2022; Sigeman et al., 2021). Within Sylvioidea, members of the Alaudidae (larks) family have two additional sex chromosome translocations involving parts of chromosomes 3 and 5 (Sigeman et al., 2019; Dierickx et al., 2020; Figure 1), and the chromosome 3 fusion is shared by their sister lineage, Panuridae (bearded reedlings; Sigeman et al., 2019; Figure 1). A further sex chromosome translocation, involving a part of chromosome 4, was discovered in the Cisticolidae (Cisticolas and allies; Sigeman et al., 2020; Figure 1).

Here, we increase our sampling within Sylvioidea to search for undetected sex chromosome diversity. We conduct whole‐genome analyses of single species‐representatives of three previously unstudied Sylvioidea families: *Sylvietta brachyura* (northern crombec) in family Macrosphenidae (African warblers; 21 species; Winkler et al., 2020a), *Pycnonotus barbatus* (common bulbul) in family Pycnonotidae (bulbuls; 151 species; Winkler et al., 2020b) and *Argya altirostris* (Iraq babbler) in family Leiothrichidae (laughing thrushes; 143 species; Winkler et al., 2020c). We discovered yet another autosome–sex chromosome fusion event within Sylvioidea, a translocation involving a part of chromosome 8 in *S. brachyura* in the Macrosphenidae family (Figure 1).

## 2 MATERIALS AND METHODS

### 2.1 Data and DNA sequencing

DNA extracted from blood samples of one male (ZZ) and one female (ZW) for each of the three studied species was sequenced with Illumina Novaseq paired‐end (2 × 150 bp) technology. Sample ID's and sampling locations of all individuals used in this study are listed in Table S1.

### 2.2 In silico identification of sex‐linked genomic regions

The paired‐end sequence files were trimmed for adaptors and low‐quality nucleotides (using nesoni clip v0.115; https://github.com/Victorian‐Bioinformatics‐Consortium/nesoni), and de novo reference genomes were constructed (using Spades v3.13.1; Prjibelski et al., 2020) based on the male (homogametic; ZZ) sample of each species (see Supplementary Methods S1.1 for details; genome assembly statistics are in Table S2). We used the pipeline findZX (Sigeman et al., 2022) to find sex‐linked genomic regions in each of the three species. Briefly, this software includes the following main steps: (i) aligning whole‐genome paired‐end data from the male and the female sample to the genome assembly of each species and (ii) scanning for genomic regions with sex‐specific differences in genome coverage and/or percentage of heterozygous sites. This general approach has been used to effectively uncover sex chromosome systems of different levels of differentiation (Darolti et al., 2019; Vicoso et al., 2013; Yoshida et al., 2014). Drastically reduced coverage, in combination with either lower or similar levels of heterozygosity, in the female compared to the male is characteristic of sex‐linked regions with high W chromosome degeneration. Genomic regions with slightly to moderately lower or similar coverage values in the female compared to the male, but clearly increased heterozygosity levels in the female, are indicative of sex‐linked regions with less degeneration (reviewed in Vicoso, 2019; Palmer et al., 2019). In contrast, autosomes, recombining parts of the sex chromosomes (i.e. pseudoautosomal regions; PARs), and parts of the sex chromosomes that evolved recombination suppression very recently, are expected to show no sex differences in either metric (genome coverage or heterozygosity).

We ran the pipeline twice for each species (see Supplementary Methods S1.2 for details). First, using the de novo reference genomes derived from the male sample of each species (see above), and second, by using the ‘consensus‐genome’ option available in findZX. This option creates a consensus reference genome from the original de novo reference genome by incorporating major alleles from both samples (based on the output of the first run), which are then used in the pipeline to equalize the mapping success between the female and male sample (coverage statistics in Table S3). The findZX pipeline also includes an option (findZX‐synteny) for anchoring the scaffold positions of the study‐species reference genome to a second, more contiguous, reference genome. We used this option to anchor the scaffolds from the genome assemblies from the studied species to the chromosome‐level assembly of *T. guttata* (taeGut3.2.4; Table S2; Warren et al., 2010). Plotting was done in R v3.6.1 (R Core Team, 2019). Throughout the Results and Discussion, we present results based on values across 100 kb genome windows, and genome coverage values that are based on filtered alignment files with maximum 2 mismatches between aligned reads and the reference genome. The Supplementary Information contains plots with both stricter (0 mismatches) and more lenient (unfiltered alignment files) mismatching stringency settings.

### 2.3 Molecular validation of novel candidate sex‐linked regions

To independently verify novel sex‐linked regions, we designed primers for PCR amplification of genome regions on chromosome 8 (based on the *S. brachyura* reference genome) and 25 (based on the *P. barbatus* reference genome). We identified exon and intron regions in each of these reference genomes by blasting the scaffolds against a database constructed from the coding sequences in the *T. guttata* gene annotation (NCBI assembly accession ID: GCF_003957565.1) using BLAST+ v2.11.0 (Camacho et al., 2009) with settings ‐max_target_seqs 1 ‐evalue 1e‐6. We designed primers (Table S4) from exon regions to capture introns in which the aligned reads from the male and female sample showed pronounced genetic differentiation. Specifically, we targeted regions where the female (ZW) was heterozygous for an insertion while the male (ZZ) was homozygous with no insertion. The expectation was thus that amplified DNA from additional females should show two bands in gel electrophoresis while additional males should show a single band (with a fragment length identical to the shorter one found in females), if the regions on chromosomes 8 and 25 were indeed sex‐linked.

In total, six *P. barbatus* individuals (three males and three females, including the genome sequenced male and female; Table S5) and five *S. brachyura* (three males and two females, of which one of the females was also used for genome sequencing; Table S5) were genotyped. All samples had been previously sexed using the primer pairs P2 and P8 (Griffiths et al., 1998). DNA from all samples were diluted to a concentration of 10–15 ng/μl, and we followed the PCR protocol described in Sigeman et al. (2020). The PCR products were separated on 3% agarose gels in TAE buffer.

### 2.4 Searching for evidence of fusion points and PAR boundaries in *Sylvietta*

Lastly, we searched for evidence of sex chromosome fusion points and PAR boundaries in the Macrosphenidae family using a published reference genome from a second *Sylvietta* species (*S. virens*, green crombec; Feng et al., 2020). The *S. virens* genome was used due to its higher contiguity (N50: 2.5 Mb; Table S2) compared to the *S. brachyura* reference genome, which was produced in this study (N50: 24 kb; Table S2). We ran the findZX pipeline on the *S. brachyura* samples (Table S1) again, this time using the *S. virens* reference genome instead of the *S. brachyura* reference genome, and both with and without *T. guttata* as a synteny‐species (Supplementary Methods S1.2). We plotted sex differences in genome coverage and heterozygosity, as well as between‐species synteny information, of chromosomes 4A and 8 using circos v0.69‐6 (Krzywinski et al., 2009). *S. virens* scaffolds showing sex differences across only parts of their length were interpreted as putative PAR boundaries, and scaffolds showing synteny with two different sex‐linked chromosomes (e.g. chromosome Z and 4A) as fusion points. Scaffolds that showed sex differences across their entire length and were positioned at the outer range of a sex‐linked region (see below), were interpreted as putative fusion points.

## 3 RESULTS AND DISCUSSION

**FIGURE 2:**
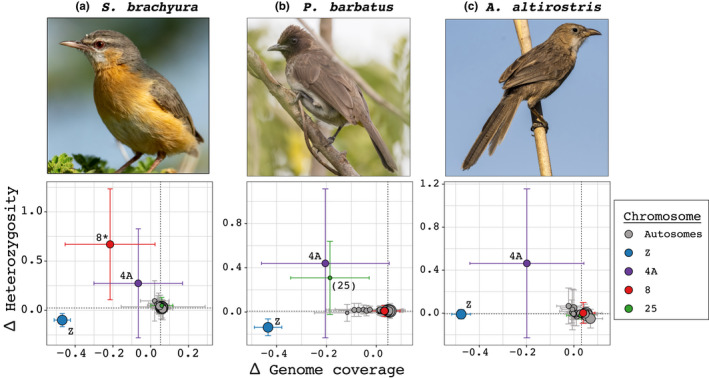
Mean (± SD) female‐to‐male difference in normalized genome coverage (*x*‐axis) and in the percentage of heterozygous sites (*y*‐axis) based on values across 100 kb windows for each chromosome in: (a) *Sylvietta brachyura* (family Macrosphenidae), (b) *Pycnonotus barbatus* (family Pycnonotidae) and (c) *Argya altirostris* (family Leiothrichidae). Chromosomes deviating strongly between the sexes in genome coverage and/or heterozygosity in any of the studied species (chromosomes Z, 4A, 8 and 25) are coloured differently from the other chromosomes. The sex‐linked signature of chromosome 8 (marked by *) in *S. brachyura* is a novel finding for this paper, which was also verified through PCR experiments using additional samples. The sex‐linked signature of chromosome 25 (in parenthesis) is also novel for this study, but PCR experiments did not find evidence of sex‐linkage for this region. The sizes of the points reflect the length of each chromosome. Each panel in Figure 2 is one of the six panels in Figures S1–S3.

The ancestral sex chromosome in all three species (chromosome Z) had substantially lower genome coverage in the females (ZW) than in the males (ZZ), in accordance with expectations of a heavily degenerated W chromosome region (mean ± SD female‐to‐male coverage‐ and heterozygosity values across 100 kb windows for each chromosome and species are given in Figure 2a–c, and additional plots including for each 100 kb window along the genome are given in Figures S1–S9). In all three species, chromosome 4A (of which a part is sex‐linked in all Sylvioidea species studied so far) had moderately lower genome coverage in the females than the males but clearly elevated heterozygosity in the females than in the males (Figures 2a–c and S1–S9). This genomic signature is indicative of a sex chromosome region with a moderately degenerated W chromosome. Two additional chromosomes deviated strongly from the autosomal pattern in both coverage and heterozygosity: chromosome 8 in *S. brachyura* (Figures 2a, S1, S4 and S5) and chromosome 25 in *P. barbatus* (Figures 2b, S2, S6 and S7). Both chromosomes had genomic signatures similar to chromosome 4A, with chromosome 8 showing more pronounced sex differences than chromosome 25.

In addition, six small chromosomes (1B, 22, 23, 26–28) in *P. barbatus* showed slightly deviating coverage values (Figures 2b and S2). However, these chromosomes showed no deviation in heterozygosity as would have been expected for relatively recently sex‐linked chromosomes, and the genome coverage standard error was significantly higher compared to the larger chromosomes (Figures S10, S11 and S12 show similar plots for the other two species). We, therefore, hypothesize that the outlier mean‐values of these six chromosomes are driven by low sample sizes (i.e. few 100 kb windows per chromosome) and do not consider them as candidate sex‐linked chromosomes.

**FIGURE 3:**
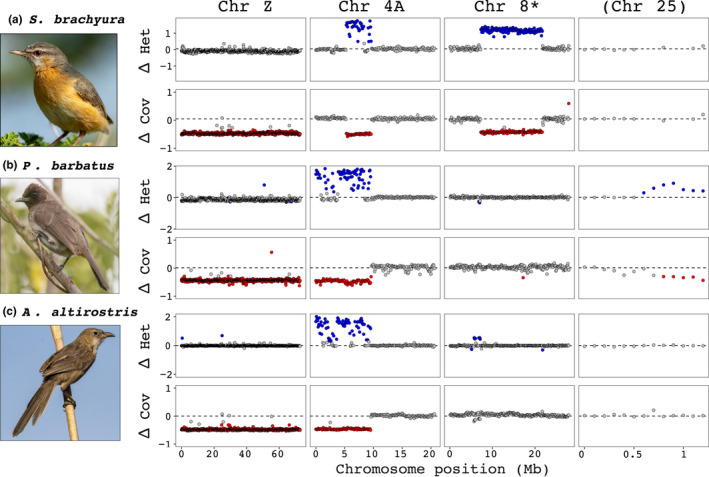
Female‐to‐male difference in heterozygosity (Het) and genome coverage (Cov) in 100 kb windows along chromosomes showing signatures of sex linkage in the studied species (chromosomes Z, 4A, 8 and 25) in (a) *Sylvietta brachyura*, (b) *Pycnonotus barbatus* and (c) *Argya altirostris*. The dashed lines mark the genome‐wide average for each species and measurement. Data points that fall beyond one standard deviation of the genome‐wide mean are coloured either blue (for heterozygosity) or red (for genome coverage). Note that the panels are not scaled for chromosome length, and that PCR experiments confirmed sex‐linkage for chromosome 8 but not for chromosome 25.

Next, we studied the four chromosomes (Z, 4A, 8 and 25) showing deviating patterns between sexes in more detail (Figure 3). Chromosome Z showed clear signs of sex‐linkage with low female‐to‐male genome coverage values across its entire length in all three species (0–72.9 Mb; Figure 3a–c), while chromosomes 4A, 8 and 25 showed signs of sex‐linkage across only parts of the chromosomes. In *P. barbatus* and *A. altirostris*, the first half of chromosome 4A (0–9.6 Mb; Figure 3b,c) showed elevated heterozygosity and drastically lower genome coverage in the female compared to the male. Thus, the entire translocated region of chromosome 4A (0–9.6 Mb; Sigeman et al., 2021; Ponnikas et al., 2022) has evolved recombination suppression in *P. barbatus* and *A. altirostris*, similar to all other previously studied Sylvioidea species (Figure 1; Dierickx et al., 2020; Leroy et al., 2021; Sigeman et al., 2019, 2020). In *S. brachyura*, however, only a part of this translocated region (5.4–9.6 Mb; Figure 3a) showed signs of sex‐linkage, whereas the remaining part (0–5.3 Mb) appears to be still recombining as it showed no sex‐specific pattern. This is an interesting observation as it suggests that the translocated region of chromosome 4A continued to recombine after the translocation event. Further support for this conclusion comes from a recent study, in which a phylogenetic analysis of Z and W gene copies from several Sylvioidea species suggests that recombination suppression evolved non‐linearly over the translocated chromosome 4A region over several million years (Sigeman et al., 2021).

In *S. brachyura* the central region of chromosome 8 (7.3–21.8 Mb) showed clear signs of sex‐linkage with higher heterozygosity and lower genome coverage in the female compared to the male (Figure 3a). The rest of chromosome 8 did not differ between sexes. PCR experiments using additional *S. brachyura* individuals confirmed sex‐linkage of the central region, as amplified DNA from all female samples but none of the male samples resulted in double bands on the gel electrophoresis for three out of the four primer pairs (the last primer pair resulted in improper annealing of PCR fragments; Figure S13). The other novel candidate sex‐linked region found in this study, on chromosome 25 (one of the smallest chromosomes) in *P. barbatus*, showed elevated female heterozygosity values across a 0.7 Mb region (0.6–1.3 Mb; Figure 3b) and low female genome coverage values across a 0.5 Mb region (0.8–1.3 Mb; Figure 3b). However, the PCR experiments using additional *P. barbatus* individuals suggest that these genetic differences are not sex‐specific, as the DNA fragments from the genome sequenced female were more similar to one of the three male samples than to the other female samples (Figure S14). Instead, it is possible that this region is polymorphic for genetically divergent, but autosomal, haplotype blocks. Future whole‐genome sequencing of additional *P. barbatus* individuals will enable us to accept or reject this hypothesis. Interestingly, chromosome 25 has fused with the Z chromosome in another bird species, *Myiopsitta monachus* (the monk parakeet; Huang et al., 2021).

**FIGURE 4:**
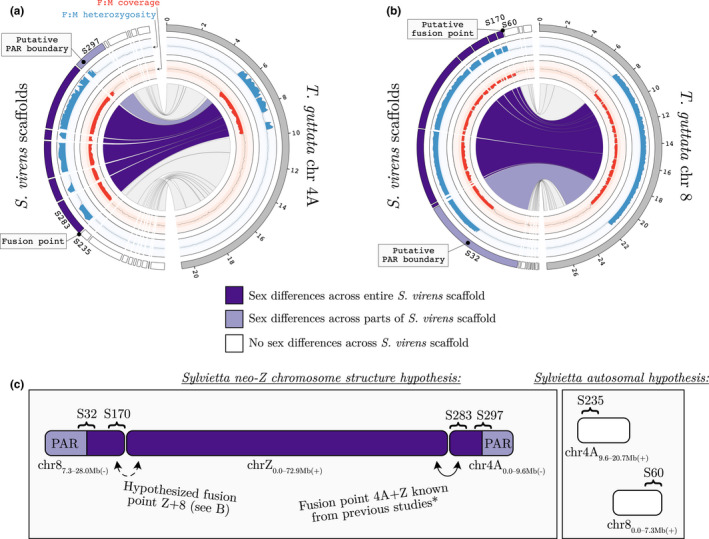
Synteny plot between *Sylvietta virens* scaffolds and *Taeniopygia guttata* chromosomes (a) 4A and (b) 8. Also shown is female‐to‐male difference in heterozygosity and genome coverage based on the *Sylvietta brachyura* samples. Scaffolds crossing, or adjacent to, non‐recombination–recombination boundaries are highlighted. (c) The hypothesized neo‐Z chromosome structure, and autosomal parts of chromosome 4A and 8, in Macrosphenidae.

Lastly, we aimed to clarify the structure of the neo‐Z chromosome in Macrosphenidae, through analyses of an available genome assembly generated by long‐read sequencing of another *Sylvietta* species, *S. virens* (Feng et al., 2020; findZX output plots are in Figures S15–S17). By projecting the *S. virens* scaffolds on the *T. guttata* chromosome‐level assembly with a synteny analysis, we found scaffolds that showed evidence of containing recombining as well as non‐recombining regions on both chromosome 4A (Scaffold297 at *T. guttata* chromosome position 5.4 Mb; Figure 4a) and chromosome 8 (Scaffold32 at position 21.8 Mb; Figure 4b). This suggests that there is a pseudoautosomal region (PAR) in each end of the *Sylvietta* neo‐Z chromosome, and thus that chromosome 4A and 8 have been fused to each end of chromosome Z, respectively. However, we found no scaffolds in *S. virens* that were crossing the (presumed) fusion points between these chromosomes and chromosome Z, as the scaffolds that were located adjacent to the possible fusion points ended where the non‐recombining regions end (Scaffold283 at *T. guttata* chromosome position 9.6 Mb for chromosome 4A and Scaffold170 at position 7.3 Mb for chromosome 8; Figure 4a,b). Thus, we cannot with the present data confirm the fusion points and the rearrangements in *Sylvietta*. Previous work on several other Sylvioidea species has, however, already confirmed that the 9.6 Mb‐end of the translocated part of chromosome 4A has fused to the 72.9 Mb‐end of chromosome Z (i.e. Z_0‐72.9(+)_–4A_9.6–0(−)_; Leroy et al., 2021; Sigeman et al., 2021) and that the remaining part of chromosome 4A segregates as an independent autosome (Ponnikas et al., 2022). Therefore, we assume that this is also the situation in *Sylvietta*. Based on the positioning of the translocated region of chromosome 4A in several Sylvioidea spp., and the finding of *S. virens* scaffolds crossing a PAR boundary on both chromosome 4A and chromosome 8, we hypothesize that the neo‐Z chromosome in Macrosphenidae spans positions 7.3–28.0 Mb of chromosome 8, the entire Z chromosome and positions 0–9.6 Mb of chromosome 4A (i.e. 8_28.0–7.3(−)_–Z_0.0–72.9(+)_–4A_9.6–0.0(−)_; Figure 4c). Future studies using cytogenetics or long‐read sequencing, and ideally chromosome‐wide genome assemblies of *Sylvietta* species, will be able to test this hypothesis.

## 4 CONCLUSION

The results from this study expand our knowledge of sex chromosome diversity in the Sylvioidea songbird system, where four autosome–sex chromosome fusions, involving chromosomes 3, 4, 4A and 5, were previously known (Dierickx et al., 2020; Leroy et al., 2021; Pala, Hasselquist, et al., 2012; Pala, Naurin, et al., 2012; Sigeman et al., 2019, 2020). We present evidence of a fifth translocation within the Sylvioidea group, involving a large part of chromosome 8 in *S. brachyura* (Figures 1, 2, 3a and 4). Interestingly, all families with confirmed multiple translocations are lineages that have diverged earlier within Sylvioidea, whereas later‐diverged lineages only carry the chromosome 4A translocation (Figure 1). The uncovered sex chromosome diversity in Sylvioidea, together with recent findings of autosome–sex chromosome fusions in four other (non‐Sylvioidea) bird lineages – *Myzomela* honeyeaters in family Meliphagidae (Burley et al., 2022; Sardell, 2016), *Eopsaltria australis* (eastern yellow robin) in family Petroicidae (Gan et al., 2019), *Crotophaga ani* (smooth‐billed ani) in family Cuculidae (Kretschmer et al., 2020) and several species in family Psittaculidae (Huang et al., 2021) – provide clear evidence for avian sex chromosomes being more evolutionary labile than previously assumed (Ellegren, 2010; Nanda et al., 2008). Sylvioidea is a species‐rich clade with over 1200 species (Fregin et al., 2012), and future studies targeting genera and families not covered by current sequencing initiatives may reveal additional sex chromosome diversity.

## AUTHOR CONTRIBUTIONS

H.S. and B.H. designed the study and wrote the manuscript. H.S. performed the bioinformatics analyses and PCR experiments. H.Z. performed the DNA extractions for the sequencing data. S.A.A. provided DNA samples for *Argya altirostris*. All authors read and approved the manuscript.

## FUNDING INFORMATION

Sequencing was performed by the SNP&SEQ Technology Platform at Uppsala Genome Center, which is part of National Genomics Infrastructure (NGI) Sweden, and Science for Life Laboratory (SciLifeLab), supported by the Swedish Research Council (and its Council for Research infrastructure, RFI) and the Knut and Alice Wallenberg Foundation. Bioinformatics analyses were performed on computational infrastructure provided by the Swedish National Infrastructure for Computing (SNIC) at Uppsala Multidisciplinary Center for Advanced Computational Science (UPPMAX). The research was funded by a grant from the Swedish Research Council (consolidator grant no. 621‐2016‐689 to B.H.).

## CONFLICT OF INTEREST

The authors have no conflict of interest to declare.

### PEER REVIEW

The peer review history for this article is available at https://publons.com/publon/10.1111/jeb.14096.

## Supporting information


Appendix S1

## Data Availability

The sequencing data used in this study are available in the NCBI Sequence Read Archive under BioProject PRJNA578893. Configuration files for the findZX analyses, along with output tables and plots, are available on Dryad (https://doi.org/10.5061/dryad.37pvmcvpb).
