# Supplementary material for: A novel neo‐sex chromosome in Sylvietta brachyura (Macrosphenidae) adds to the extraordinary avian sex chromosome diversity among Sylvioidea songbirds
Source: J Evol Biol. 2022 Sep 26;35(12):1797–805. doi: 10.1111/jeb.14096 (PMC10087220; doi:10.1111/jeb.14096)
Supplement: Supplementary file 1 — Appendix S1 [file JEB-35-1797-s001.pdf]

Supplementary Information

A novel neo-sex chromosome in *Sylvietta brachyura* (Macrosphenidae) adds to the extraordinary avian sex chromosome diversity among Sylvioidea songbirds

1 Supplementary Methods

1.1 Creating *de novo* reference genomes

We created *de novo* reference genomes for the male sample of each species (Table S1). First, the raw fastq files were trimmed using nesoni v0.115 (https://github.com/Victorian-Bioinformatics-Consortium/nesoni) using the following options:

```
python -m nesoni clip: clipped --out-separate yes --gzip no --quality 20 \
--length 20 pairs: <forward_reads>.fastq.gz <reverse_reads>.fastq.gz
```

Then, we used spades v3.13.1 [1] to create reference genomes from the trimmed data:

```
spades.py -k 21,33,55,77,99,127 --pe1-1 <forward_reads_trimmed>.fq \
--pe1-2 <forward_reads_trimmed>.fq -o spades_output -t 20 -m 256
```

1.2 FindZX analyses

We used the findZX pipeline [2] to identify sex-linked regions in the three species. Configuration files, sample information files, and all output tables and plots are available on Dryad (doi:10.5061/dryad.37pvmcvpb).

We ran the findZX pipeline on all three species:

```
snakemake -s workflow/findZX-synteny --cores 20 -R all --configfile \
config/Pycnonotus_barbatus_config.yml --use-conda -k
```

```
snakemake -s workflow/findZX-synteny --cores 20 -R all --configfile \
config/Sylvietta_brachyura_config.yml --use-conda -k
```

```
snakemake -s workflow/findZX-synteny --cores 20 -R all --configfile \
config/Turdoides_altirostris_config.yml --use-conda -k
```

Then, we created consensus reference genomes (through the same pipeline):

```
snakemake -s workflow/findZX-synteny --cores 1 -R modify_genome \
--configfile config/Pycnonotus_barbatus_config.yml --use-conda -k
```

```
snakemake -s workflow/findZX-synteny --cores 1 -R modify_genome \
--configfile config/Sylvietta_brachyura_config.yml --use-conda -k
```

```
snakemake -s workflow/findZX-synteny --cores 1 -R modify_genome \
--configfile config/Turdoides_altirostris_config.yml --use-conda -k
```

Then, we ran the findZX pipeline again using these consensus reference genomes:

```
snakemake -s workflow/findZX-synteny --cores 20 -R all --configfile \
config/Pycnonotus_barbatus_consensus_config.yml --use-conda -k
```

```
snakemake -s workflow/findZX-synteny --cores 20 -R all --configfile \
config/Sylvietta_brachyura_consensus_config.yml --use-conda -k
```

```
snakemake -s workflow/findZX-synteny --cores 20 -R all --configfile \
config/Turdoides_altirostris_consensus_config.yml --use-conda -k
```

Lastly, we ran the findZX pipeline (both with and without *Taeniopygia guttata* (Table S2) as a synteny-species) for *Sylvietta brachyura* samples (Table S1) using the *Sylvietta virens* (Table S2) reference genome:

```
snakemake -s workflow/findZX --cores 20 -R all \
--configfile config/Sylvietta_brachyura_virens_config.yml --use-conda -k
```

```
snakemake -s workflow/findZX-synteny --cores 20 -R all \
--configfile config/Sylvietta_brachyura_virens_config.yml --use-conda -k
```

2 Supplementary Tables

Table S1: Information on genome sequenced samples used for this study. The sequencing data used in this study are available in the NCBI Sequence Read Archive under Bioproject PRJNA578893.

Species	Common name	Sex	Heterogametic/homogametic	Sample ID	Sampling location
Argya altirostris	Iraq babbler	female	Heterogametic	IB-2b_S32	Iraq (Al-Qadisiyah)
Argya altirostris	Iraq babbler	male	Homogametic	IB-1a_S31	Iraq (Al-Qadisiyah)
Sylvietta brachyura	Northern Crombec	female	Heterogametic	Sbra-553_S28	Nigeria (Malamfatori)
Sylvietta brachyura	Northern Crombec	male	Homogametic	Sbra-878_S26	Nigeria (Malamfatori)
Pycnonotus barbatus	Common bulbul	female	Heterogametic	Pbar-197_S24	Nigeria (Amurum)
Pycnonotus barbatus	Common bulbul	male	Homogametic	Pbar-421_S22	Nigeria (Amurum)

Table S2: Reference genome information. N50 and total length calculated with assembly-stats v1.0.1 (https://github.com/sanger-pathogens/assembly-stats).

Species	Reference genome	N50 (bp)	Total length (bp)	Sample ID	This study
Argya altirostris	Argya_altirostris	112977	1072337959	IB-1a_S31	yes
Argya altirostris	Argya_altirostris_nonRefAf_consensus	112975	1072338995	IB-1a_S31	yes
Sylvietta brachyura	Sylvietta_brachyura	23515	1135363362	Sbra-878_S26	yes
Sylvietta brachyura	Sylvietta_brachyura_nonRefAf_consensus	23518	1135388064	Sbra-878_S26	yes
Pycnonotus barbatus	Pycnonotus_barbatus	57880	1069613391	Pbar-421_S22	yes
Pycnonotus barbatus	Pycnonotus_barbatus_nonRefAf_consensus	57886	1069733087	Pbar-421_S22	yes
Sylvietta virens	GCA_013399515.1 (GenBank accession)	2514434	1028027743	B10K-DU-009-59	no
Taeniopygia guttata	GCF_000151805.1 (GenBank accession)	73657157	1233186341	Black17	no

Table S3: Average genome coverage values per sample and reference genome (calculated as the number of aligned basepairs divided by the length of the reference genome).

			Average genome coverage		
Species	Sample ID	Reference genome	Unfiltered	≤2 mismatches	0 mismatches
Argya altirostris	IB-2b_S32	Argya_altirostris	29.0932	28.3314	24.249
Argya altirostris	IB-1a_S31	Argya_altirostris	37.7751	37.2653	33.1515
Sylvietta brachyura	Sbra-553_S28	Sylvietta_brachyura	24.686	20.104	10.347
Sylvietta brachyura	Sbra-878_S26	Sylvietta_brachyura	23.4667	21.8141	16.4062
Pycnonotus barbatus	Pbar-197_S24	Pycnonotus_barbatus	38.2324	34.043	18.373
Pycnonotus barbatus	Pbar-421_S22	Pycnonotus_barbatus	37.0542	35.2467	26.7468
Argya altirostris	IB-2b_S32	Argya_altirostris_nonRefAf_consensus	29.214	28.5735	25.1865
Argya altirostris	IB-1a_S31	Argya_altirostris_nonRefAf_consensus	37.9105	37.2966	32.5978
Sylvietta brachyura	Sbra-553_S28	Sylvietta_brachyura_nonRefAf_consensus	24.866	21.9287	14.1302
Sylvietta brachyura	Sbra-878_S26	Sylvietta_brachyura_nonRefAf_consensus	23.6541	21.3512	13.0953
Pycnonotus barbatus	Pbar-197_S24	Pycnonotus_barbatus_nonRefAf_consensus	38.3184	35.5895	23.9954
Pycnonotus barbatus	Pbar-421_S22	Pycnonotus_barbatus_nonRefAf_consensus	37.1313	34.9728	23.4504
Sylvietta brachyura	Sbra-553_S28	Sylvietta_virens	27.5955	8.80688	1.64402
Sylvietta brachyura	Sbra-878_S26	Sylvietta_virens	26.0923	8.33851	1.54853

Table S4: Information on primers used to verify novel candidate sex-linked regions (see Main text for details). Primer names that start with the same numbers (e.g., 15508_F and 15508_R) are forward and reverse primers of the same pair.

Primer name	Sequence (5'-3')	Chromosome (based on synteny to *T. guttata*)	Designed from reference genome (see Table S2)	Primer designed from genome region
15508_F	TGAGGTCCCCATCAGTGTTG	25	Pycnonotus_barbatus	NODE_15508_length_17449_cov_3.264923:15,523-16,220
15508_R	CCCGGGATGTTGCGAAATTC	25	Pycnonotus_barbatus	NODE_15508_length_17449_cov_3.264923:15,523-16,220
10386_F	TCAGCTCGTTCCACTCCTTC	25	Pycnonotus_barbatus	NODE_10386_length_29521_cov_3.099782:11,255-11,897
10386_R	GCAGACGCAGCAGAACAAG	25	Pycnonotus_barbatus	NODE_10386_length_29521_cov_3.099782:11,255-11,897
7667_F	AGTGGCACAACTTCATCCCA	8	Sylvietta_brachyura	NODE_7667_length_30884_cov_2.074227:628-885
7667_R	CACGCCCCGGCTCTTCTC	8	Sylvietta_brachyura	NODE_7667_length_30884_cov_2.074227:628-885
18234_F	CTCTAAGGCCAGGGACAGGT	8	Sylvietta_brachyura	NODE_18234_length_15359_cov_2.142660:12,863-13,761
18234_R	AGAGGAAAATTGCCTTGAAATTTCA	8	Sylvietta_brachyura	NODE_18234_length_15359_cov_2.142660:12,863-13,761
12981_F	CACAAGTAGTGCTGAGGTCCA	8	Sylvietta_brachyura	NODE_12981_length_20820_cov_1.998647:3,405-4,004
12981_R	GCTGCTGAATGTCCCATAGC	8	Sylvietta_brachyura	NODE_12981_length_20820_cov_1.998647:3,405-4,004
951_F	GGTCTTCGGATGAGAAGGAGC	8	Sylvietta_brachyura	NODE_951_length_96028_cov_2.274929:48,394-49,058
951_R	GTCATAGAGGCTGCTCAGGAG	8	Sylvietta_brachyura	NODE_951_length_96028_cov_2.274929:48,394-49,058

Table S5: Samples used for PCR, and DNA concentrations (ng/µl). Samples which were also used for WGS sequencing (in this study) are indicated in the last column.

Species	Sample	Sex	DNA conc.	WGS (see Table 1)
Pycnonotus barbatus	P.bar_197	female	12.8	yes (Pbar-197_S24)
Pycnonotus barbatus	P.bar_219	female	11.2	no
Pycnonotus barbatus	P.bar_421	male	12.9	yes (Pbar-421_S22)
Pycnonotus barbatus	P.bar_428	male	13.7	no
Pycnonotus barbatus	P.bar_642	female	12.0	no
Pycnonotus barbatus	P.bar_663	male	14.0	no
Sylvietta brachyura	S.bra_141	male	11.7	no
Sylvietta brachyura	S.bra_538	female	11.1	no
Sylvietta brachyura	S.bra_553	female	12.6	yes (Sbra-553_S28)
Sylvietta brachyura	S.bra_558	male	10.3	no
Sylvietta brachyura	S.bra_560	male	11.2	no

3 Supplementary Figures

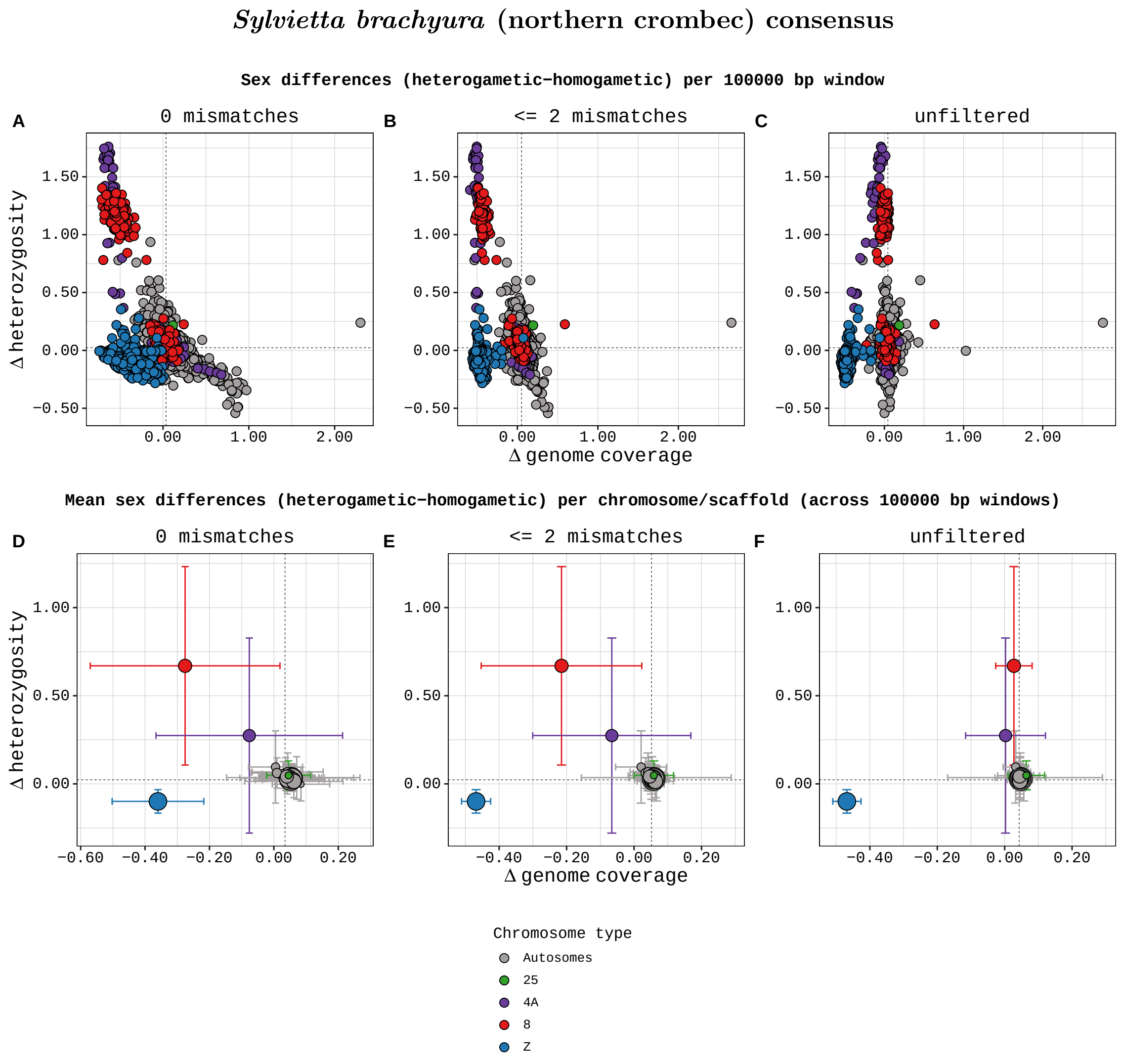

Figure S1: **(A-C)** Sex differences (female-male) in genome coverage and heterozygosity for all 100 kb genome windows. **(D-F)** Mean (± SD) sex differences in genome coverage and heterozygosity per chromosome/scaffold, calculated from the 100 kb genome windows. Dashed lines mark the genome-wide median across all 100 kb windows. Data from 1 male and 1 female *S. brachyura*, analysed using findZX-synteny.

Pycnonotus barbatus (common bulbul) consensus

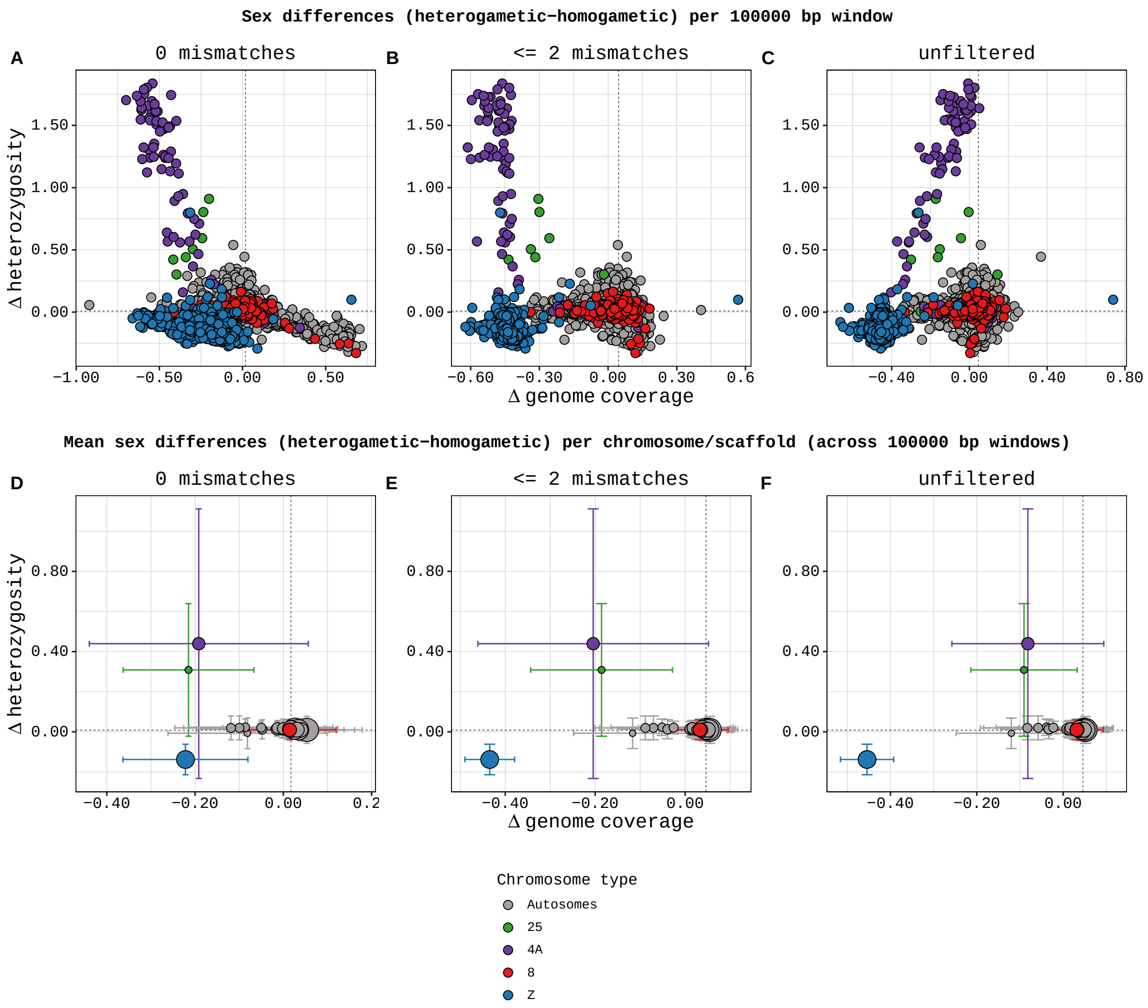

Figure S2: **(A-C)** Sex differences (female-male) in genome coverage and heterozygosity for all 100 kb genome windows. **(D-F)** Mean (± SD) sex differences in genome coverage and heterozygosity per chromosome/scaffold, calculated from the 100 kb genome windows. Dashed lines mark the genome-wide median across all 100 kb windows. Data from 1 male and 1 female *P. barbatus*, analysed using findZX-synteny.

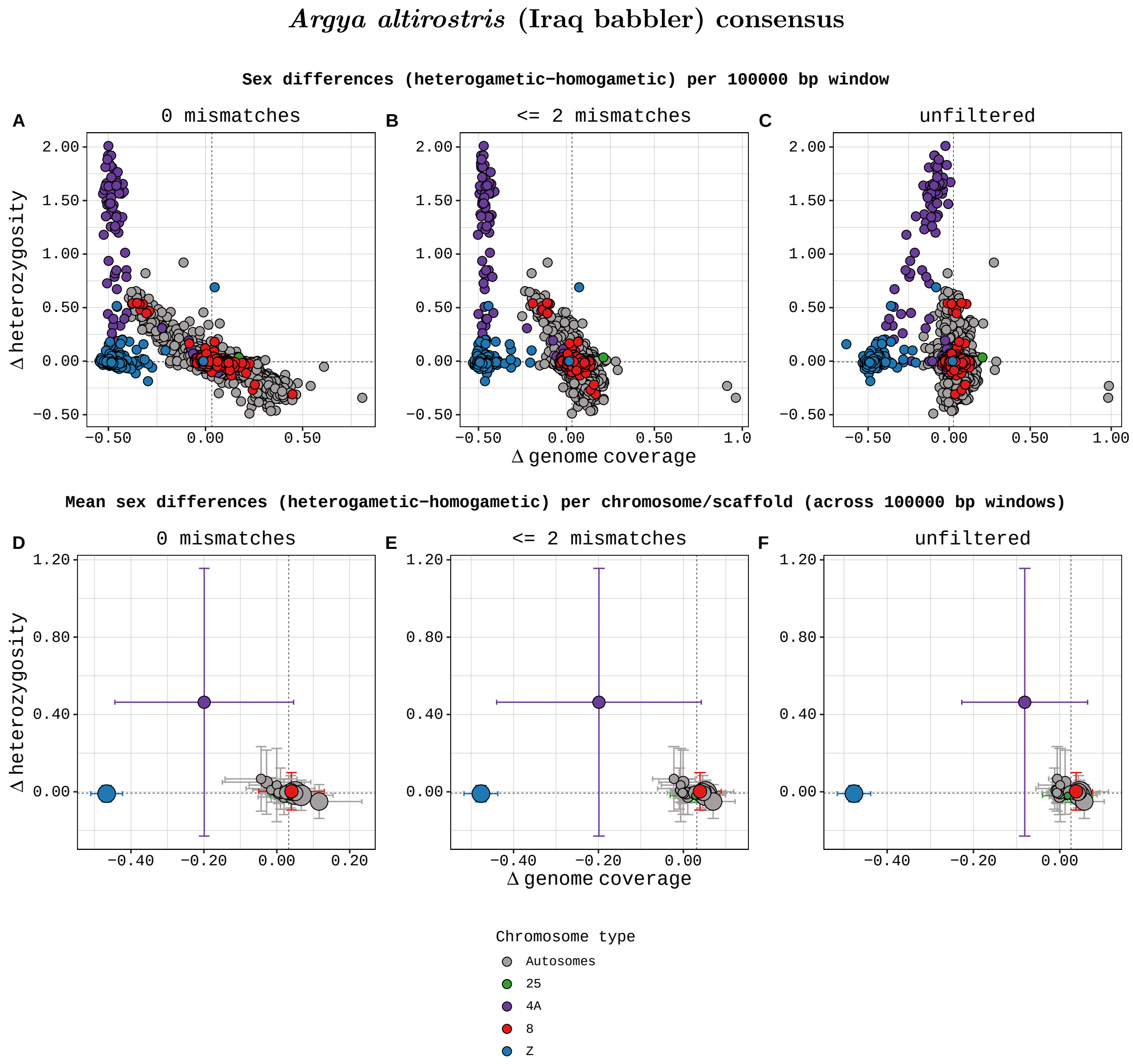

Figure S3: **(A-C)** Sex differences (female-male) in genome coverage and heterozygosity for all 100 kb genome windows. **(D-F)** Mean (± SD) sex differences in genome coverage and heterozygosity per chromosome/scaffold, calculated from the 100 kb genome windows. Dashed lines mark the genome-wide median across all 100 kb windows. Data from 1 male and 1 female *A. altirostris*, analysed using findZX-synteny.

Sylvietta brachyura (northern crombec) consensus

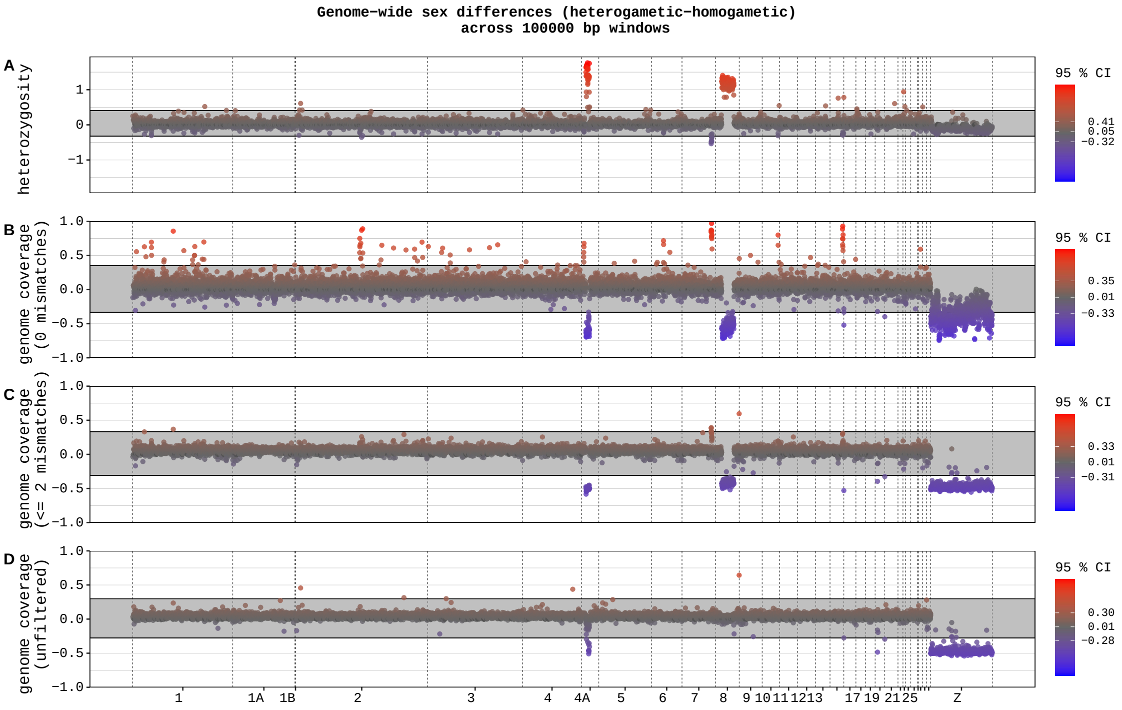

Figure S4: Sex differences (female-male) in genome coverage and heterozygosity (100 kb windows) for *S. brachyura*, plotted along chromosome positions in the *T. guttata* genome. The four rows show: (**A**) heterozygosity, and genome coverage with (**B**) strict filtering (0 mismatches allowed), (**C**) intermediate filtering (≤2 mismatches) and (**D**) no filtering of mapped reads ("unfiltered").

***Sylvietta brachyura* (northern crombec) consensus**

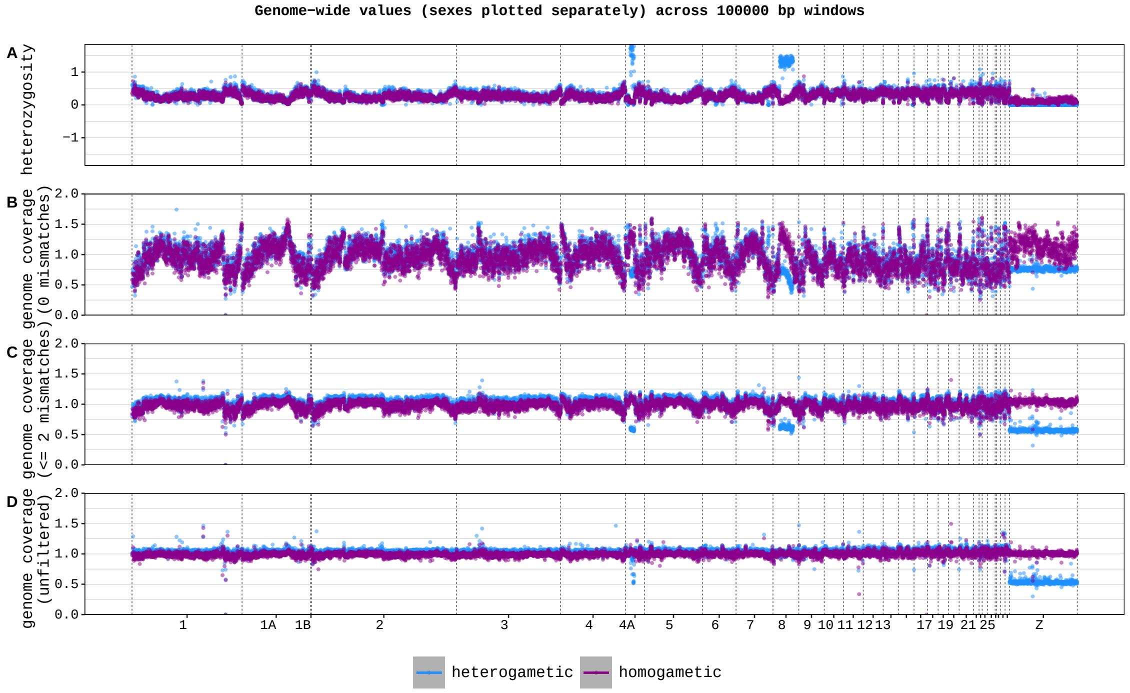

Figure S5: Per-sex genome coverage and heterozygosity values (100 kb windows) for *S. brachyura*, plotted along chromosome positions in the *T. guttata* genome. The four rows show: (**A**) heterozygosity, and genome coverage with (**B**) strict filtering (0 mismatches allowed), (**C**) intermediate filtering ($\leq$2 mismatches) and (**D**) no filtering of mapped reads ("unfiltered"). The values for each sex are plotted separately, with a smoothing line for each sex (heterogametic in blue, homogametic in purple).

Pycnonotus barbatus **(common bulbul) consensus**

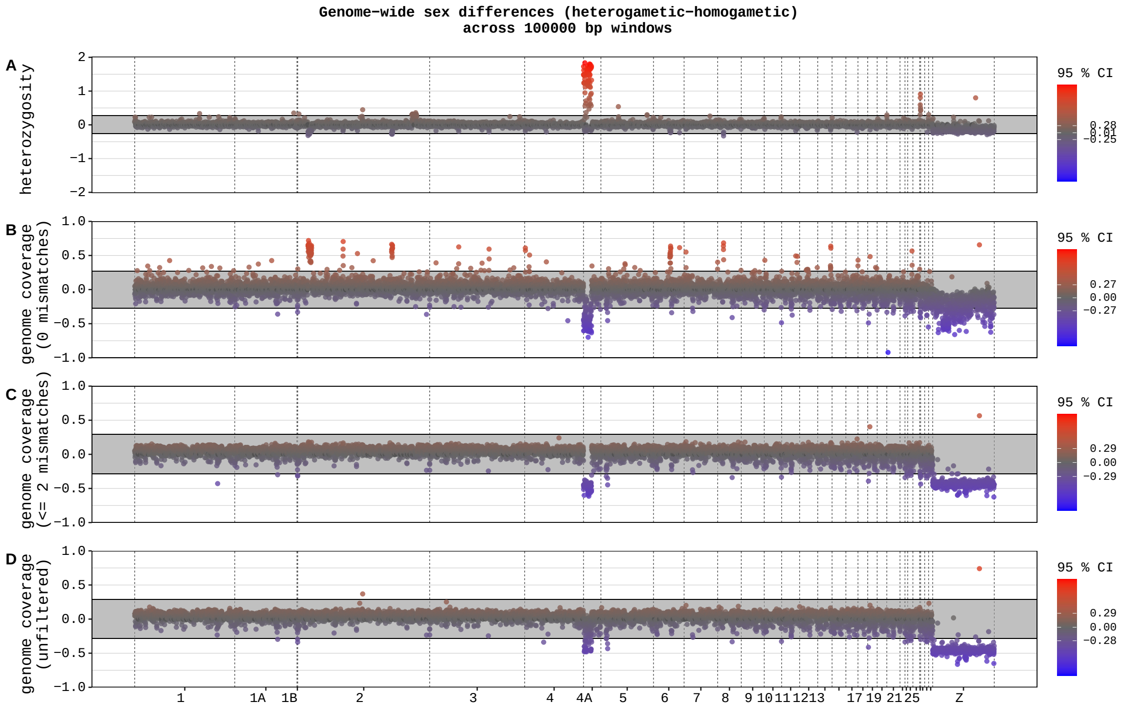

Figure S6: Sex differences (female-male) in genome coverage and heterozygosity (100 kb windows) for *P. barbatus*, plotted along chromosome positions in the *T. guttata* genome. The four rows show: (**A**) heterozygosity, and genome coverage with (**B**) strict filtering (0 mismatches allowed), (**C**) intermediate filtering ($\leq$2 mismatches) and (**D**) no filtering of mapped reads ("unfiltered").

Pycnonotus barbatus **(common bulbul) consensus**

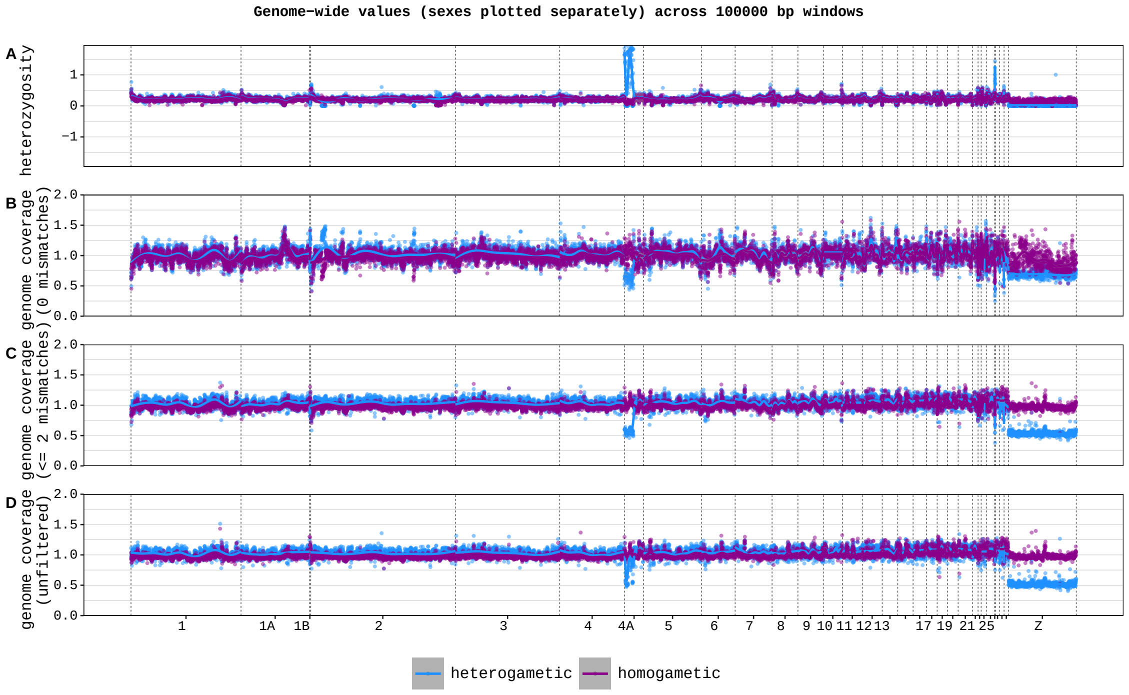

Figure S7: Per-sex genome coverage and heterozygosity values (100 kb windows) for *P. barbatus*, plotted along chromosome positions in the *T. guttata* genome. The four rows show: (**A**) heterozygosity, and genome coverage with (**B**) strict filtering (0 mismatches allowed), (**C**) intermediate filtering (≤2 mismatches) and (**D**) no filtering of mapped reads ("unfiltered"). The values for each sex are plotted separately, with a smoothing line for each sex (heterogametic in blue, homogametic in purple).

Argya altirostris (Iraq babbler) consensus

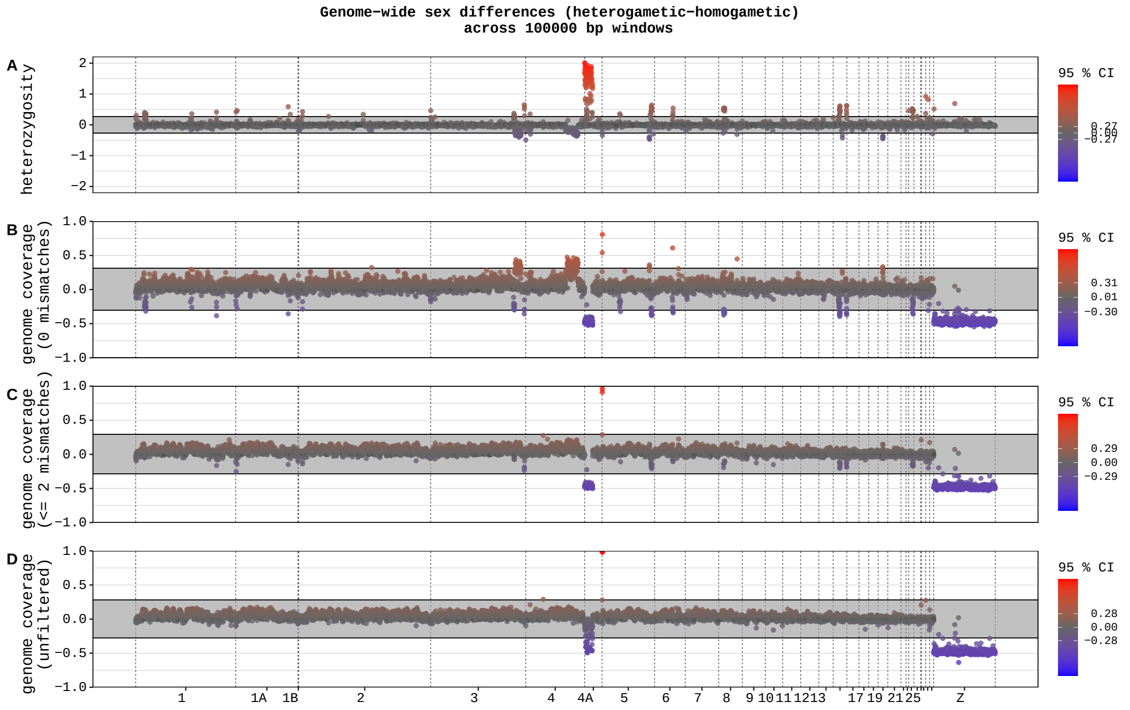

Figure S8: Sex differences (female-male) in genome coverage and heterozygosity (100 kb windows) for *A. altirostris*, plotted along chromosome positions in the *T. guttata* genome. The four rows show: (**A**) heterozygosity, and genome coverage with (**B**) strict filtering (0 mismatches allowed), (**C**) intermediate filtering (≤2 mismatches) and (**D**) no filtering of mapped reads ("unfiltered").

Argya altirostris (Iraq babbler) consensus

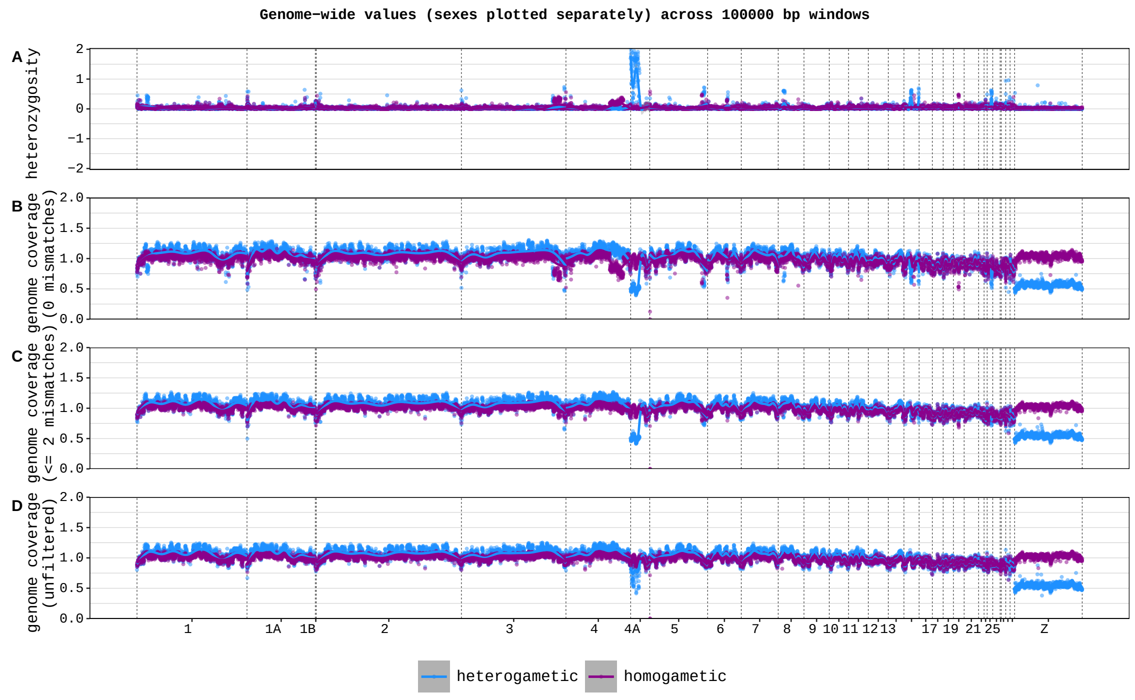

Figure S9: Per-sex genome coverage and heterozygosity values (100 kb windows) for *A. altirostris*, plotted along chromosome positions in the *T. guttata* genome. The four rows show: (**A**) heterozygosity, and genome coverage with (**B**) strict filtering (0 mismatches allowed), (**C**) intermediate filtering (≤2 mismatches) and (**D**) no filtering of mapped reads ("unfiltered"). The values for each sex are plotted separately, with a smoothing line for each sex (heterogametic in blue, homogametic in purple).

***Pycnonotus barbatus* (common bulbul) consensus**

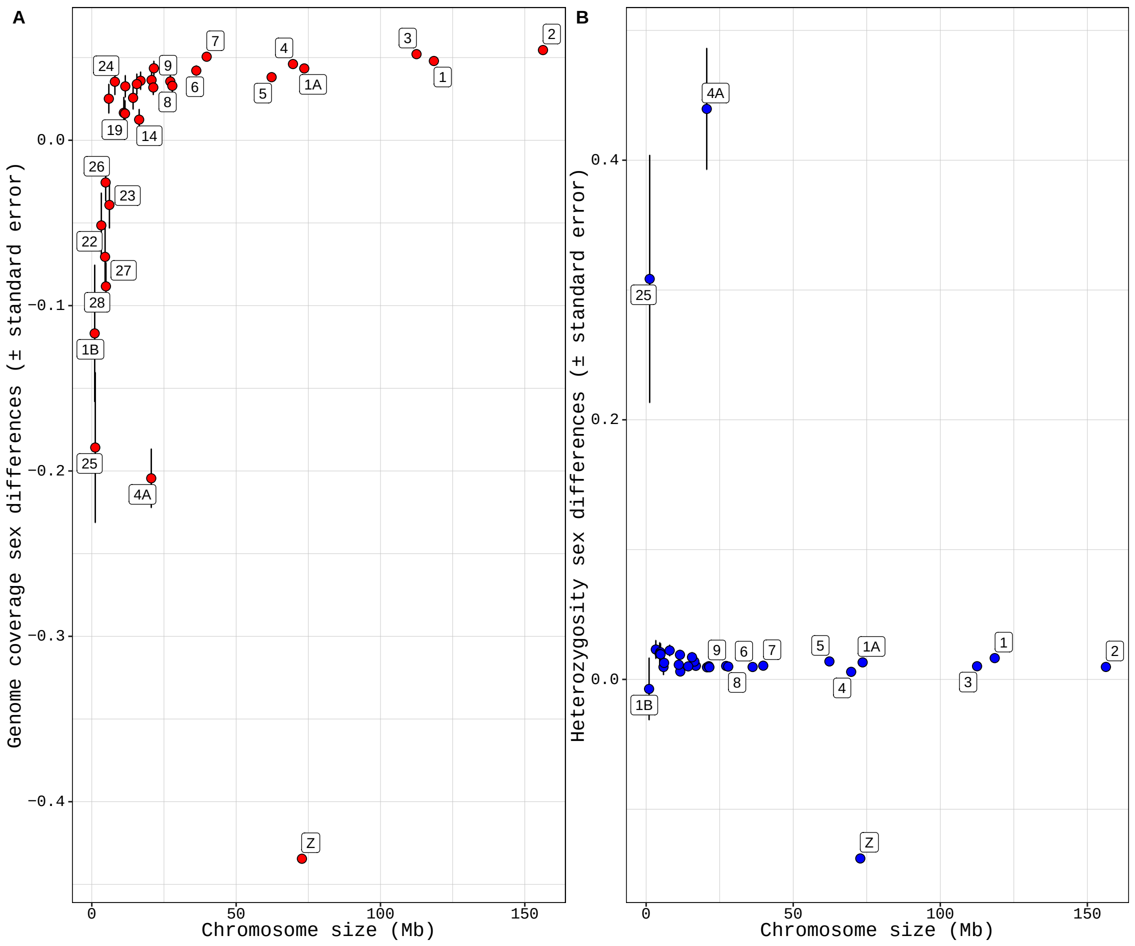

Figure S10: Mean (**a**) genome coverage and (**b**) heterozygosity values per chromosome (y-axis), plotted against the chromosome size (x-axis). The vertical lines extend to the standard error from the mean.

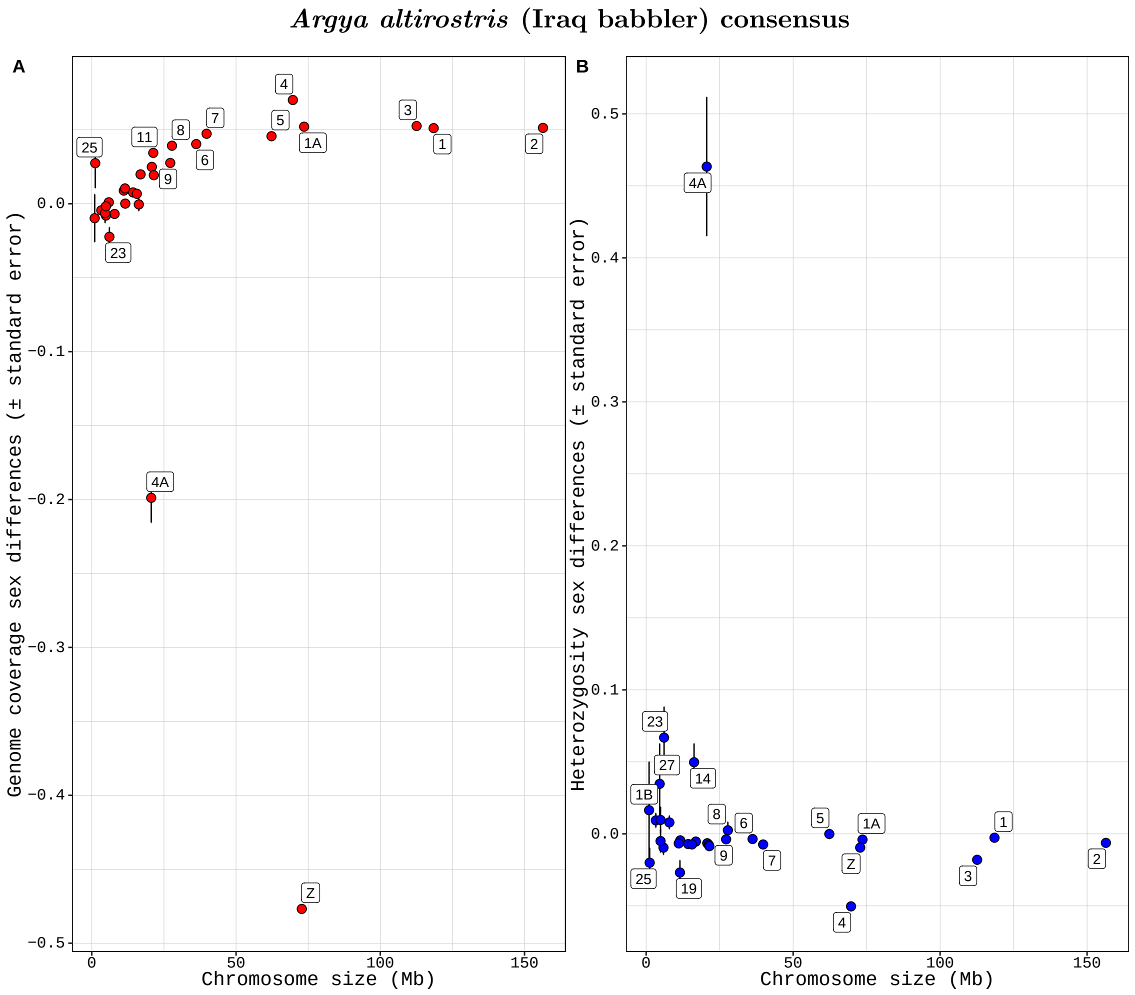

Figure S11: Mean (**a**) genome coverage and (**b**) heterozygosity values per chromosome (y-axis), plotted against the chromosome size (x-axis). The vertical lines extend to the standard error from the mean.

Sylvietta brachyura (northern crombec) consensus

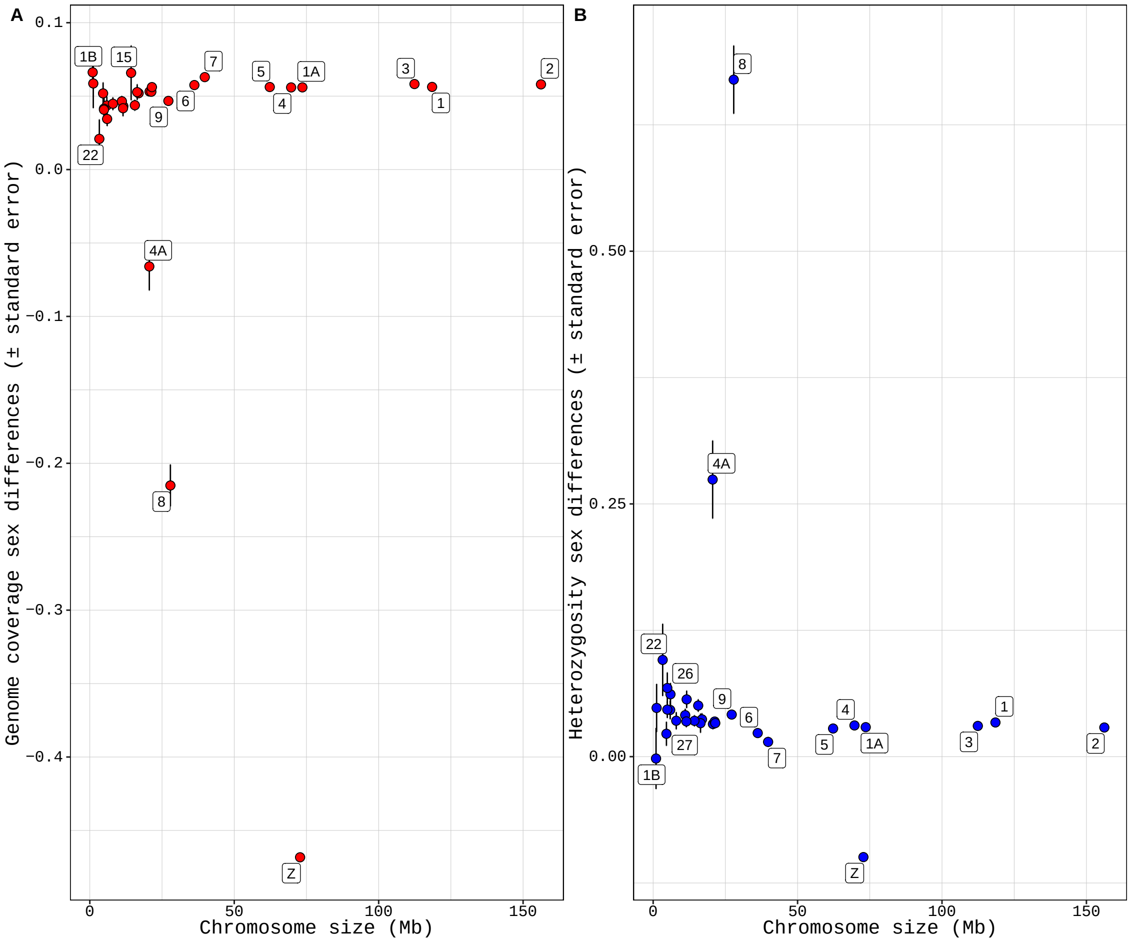

Figure S12: Mean (**a**) genome coverage and (**b**) heterozygosity values per chromosome (y-axis), plotted against the chromosome size (x-axis). The vertical lines extend to the standard error from the mean.

Gel electrophoresis image - *Sylvietta brachyura* (northern crombec)

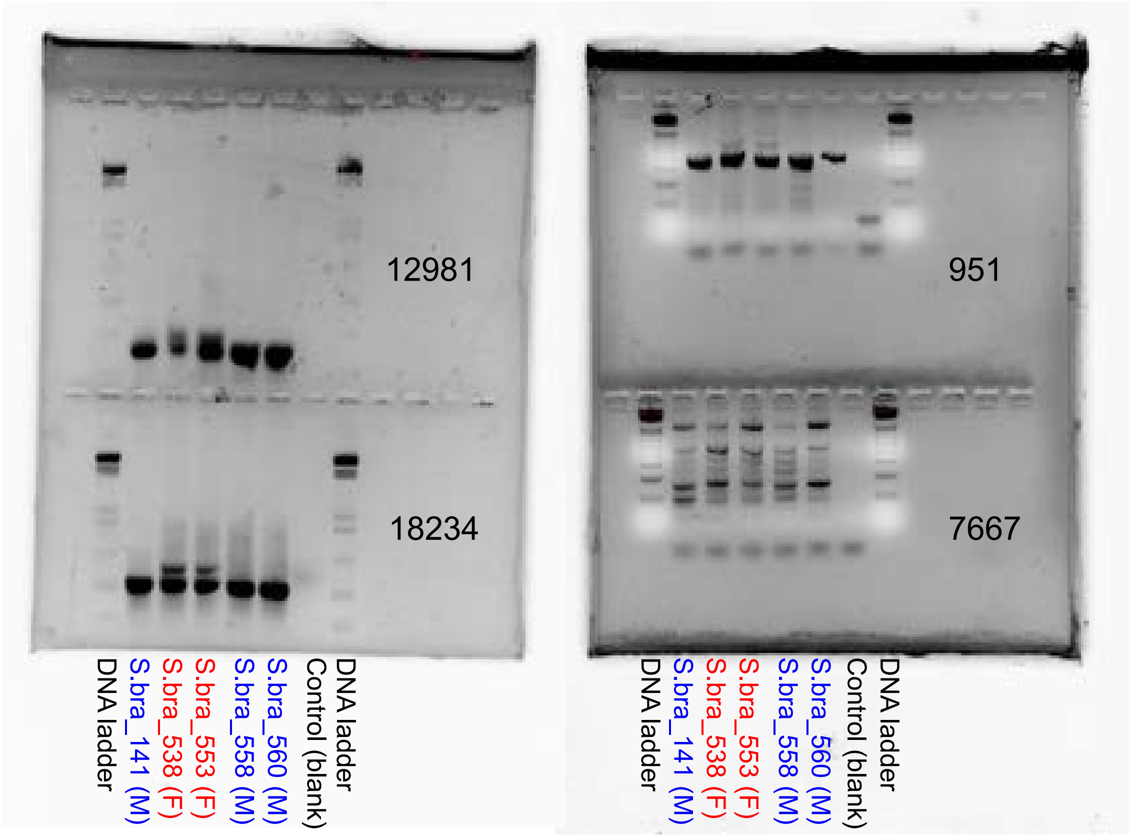

Figure S13: Gel electrophoresis image showing amplified DNA fragments from *S. brachyura* samples. The numbers on the gels indicate the primer pair (Table S4). The sample ID's (Table S5) and sex (F = female, M = male) is marked below each gel. The primer pairs were designed to amplify intron regions where the sequenced female, but not the male, was heterozygous for an insertion (see Materials & Methods). We hypothesized that these insertions are positioned on the W chromosome, and thus that other females should carry the same insertions. As expected, both females show double bands (indicating DNA fragments of different lengths) while all males show single bands for three of the four primer pairs (although clearest for primer pair 18234). The last primer pair (7667) show signs of improper annealing of PCR fragments. Note that the DNA fragment uniquely amplified in the female samples have travelled a shorter distance on the gel (i.e., it is longer than the DNA fragment amplified in the males), which is expected from a female-specific insertion.

Gel electrophoresis image - *Pycnonotus barbatus* (common bulbul)

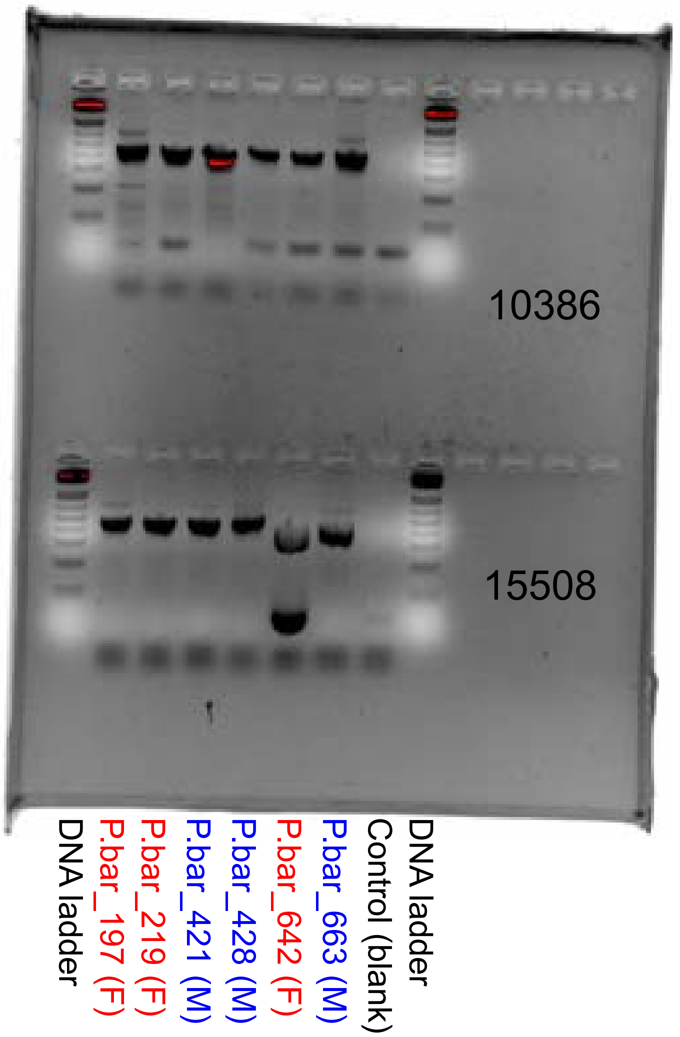

Figure S14: Gel electrophoresis image showing amplified DNA fragments from *P. barbatus* samples. The numbers on the gels indicate the primer pair (Table S4). The sample ID's (Table S5) and sex (F = female, M = male) is marked below each gel. The primer pairs were designed to amplify intron regions where the sequenced female, but not the male, was heterozygous for an insertion (see Materials & Methods). We hypothesized that these insertions are positioned on the W chromosome, and thus that other females should carry the same insertions. However, we did not find support for this hypothesis from these PCR experiments. Both DNA fragments were amplified in the genome sequenced female (P.bar_197), although the longer DNA fragment was only weakly amplified. The other females, however, only showed amplification of the lower of the two bands found in the genome sequenced female. Instead, the genome sequenced female (P.bar_197) show similar band patterns as one of the males (P.bar_663). Based on these results, we suggest that this genome region is not sex-linked.

Sylvietta brachyura (northern crombec) with *Sylvietta virens* (green crombec) genome

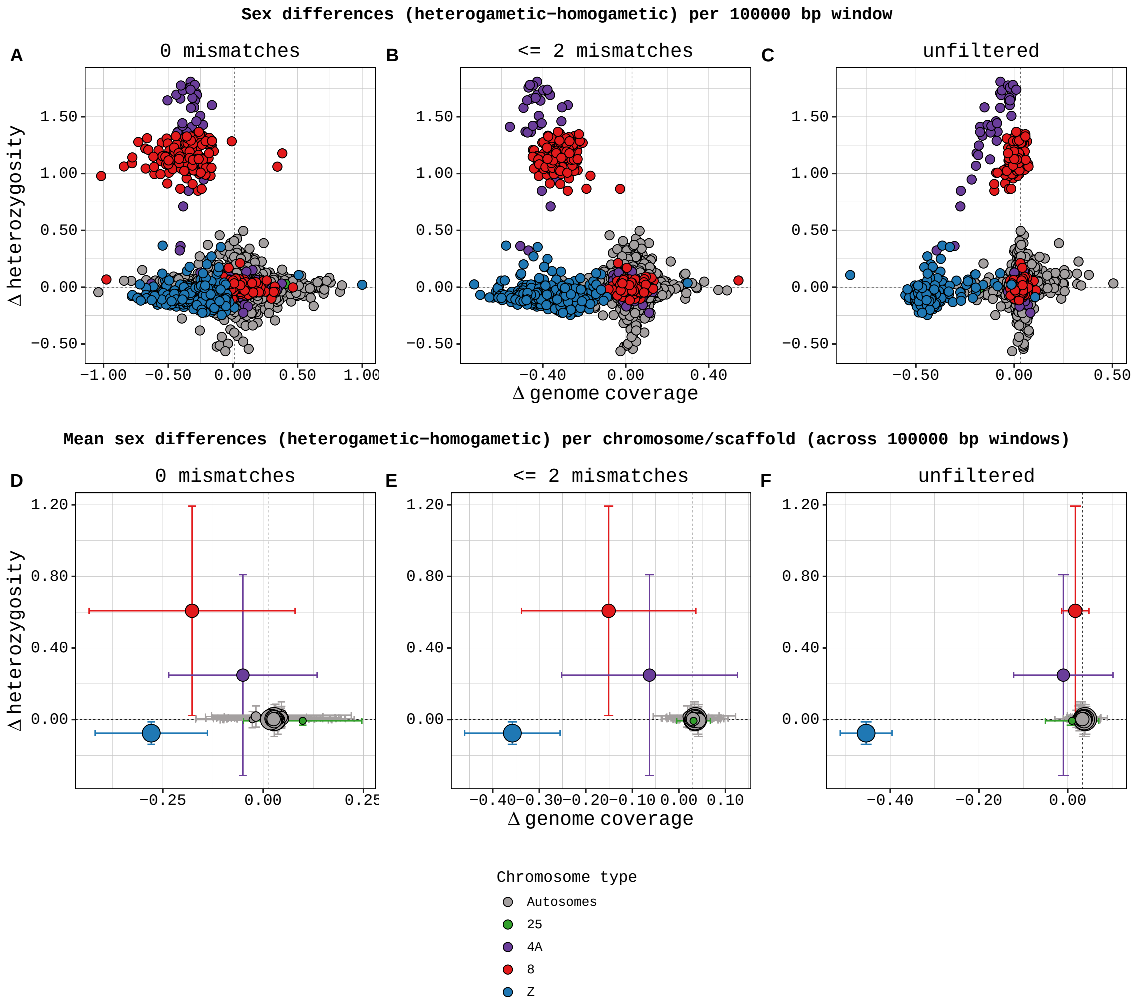

Figure S15: **(A-C)** Sex differences (female-male) in genome coverage and heterozygosity for all 100 kb genome windows. **(D-F)** Mean (± SD) sex differences in genome coverage and heterozygosity per chromosome/scaffold, calculated from the 100 kb genome windows. Dashed lines mark the genome-wide median across all 100 kb windows. Data from 1 male and 1 female *S. brachyura*, analysed using findZX-synteny

Sylvietta brachyura (northern crombec) with *Sylvietta virens* (green crombec) genome

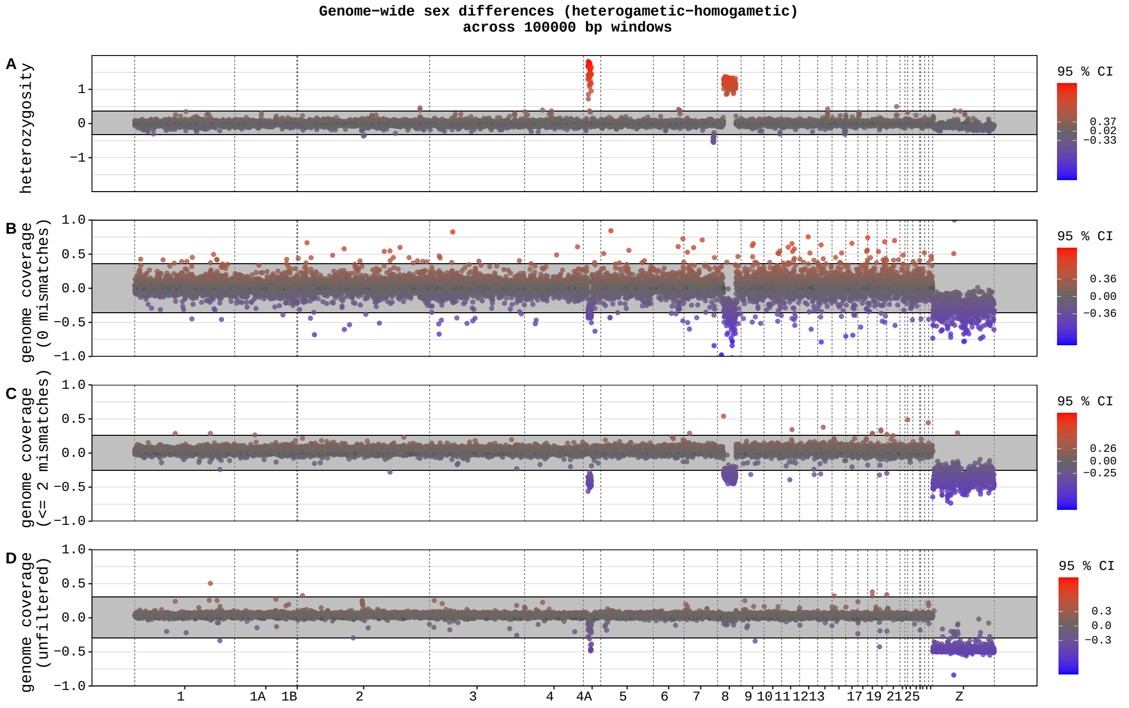

Figure S16: Sex differences (female-male) in genome coverage and heterozygosity (100 kb windows) for *S. brachyura*, plotted along chromosome positions in the *T. guttata* genome. The four rows show: (**A**) heterozygosity, and genome coverage with (**B**) strict filtering (0 mismatches allowed), (**C**) intermediate filtering (≤2 mismatches) and (**D**) no filtering of mapped reads ("unfiltered").

Sylvietta brachyura (northern crombec) with *Sylvietta virens* (green crombec) genome

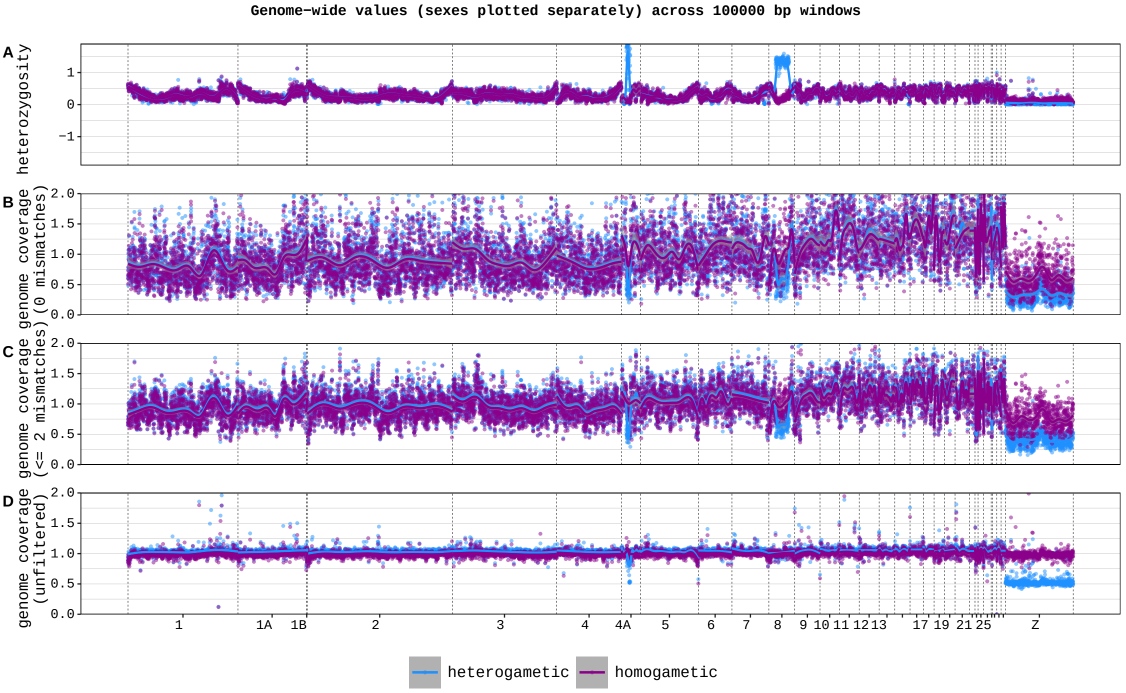

Figure S17: Per-sex genome coverage and heterozygosity values (100 kb windows) for *S. brachyura*, plotted along chromosome positions in the *T. guttata* genome. The four rows show: (**A**) heterozygosity, and genome coverage with (**B**) strict filtering (0 mismatches allowed), (**C**) intermediate filtering ($\leq$2 mismatches) and (**D**) no filtering of mapped reads ("unfiltered"). The values for each sex are plotted separately, with a smoothing line for each sex (heterogametic in blue, homogametic in purple).

References

[1] Prjibelski A, Antipov D, Meleshko D, Lapidus A, Korobeynikov A. Using SPAdes De Novo Assembler. *Curr Protoc Bioinformatics.* 2020 Jun;70(1):e102. doi: 10.1002/cpbi.102. PMID: 32559359.

[2] Sigeman H, Sinclair B, Hansson B. Findzx: an automated pipeline for detecting and visualising sex chromosomes using whole-genome sequencing data. BMC Genomics. 2022 Apr 27;23(1):328. doi: 10.1186/s12864-022-08432-9. PMID: 35477344; PMCID: PMC9044604.
